# Morphology-Dependent Catalytic Activity of Ru/CeO_2_ in Dry Reforming of Methane

**DOI:** 10.3390/molecules24030526

**Published:** 2019-02-01

**Authors:** Lulu He, Yuanhang Ren, Yingyi Fu, Bin Yue, Shik Chi Edman Tsang, Heyong He

**Affiliations:** 1 Department of Chemistry and Shanghai Key Laboratory of Molecular Catalysis and Innovative Materials, Collaborative Innovation Center of Chemistry for Energy Materials, Fudan University, Shanghai 200438, China; 14110220027@fudan.edu.cn (L.H.); yuanhangren@fudan.edu.cn (Y.R.); 13110220046@fudan.edu.cn (Y.F.); 2 Wolfson Catalysis Centre, Department of Chemistry, University of Oxford, Oxford OX1 3QR, UK; edman.tsang@chem.ox.ac.uk

**Keywords:** carbon dioxide, methane, CeO_2_, crystal facets, dry reforming

## Abstract

Three morphology-controlled CeO_2_, namely nanorods (NRs), nanocubes (NCs), and nanopolyhedra (NPs), with different mainly exposed crystal facets of (110), (100), and (111), respectively, have been used as supports to prepare Ru (3 wt.%) nanoparticle-loaded catalysts. The catalysts were characterized by H_2_-temperature programmed reduction (H_2_-TPR), CO– temperature programmed desorption (CO-TPD), N_2_ adsorption–desorption, X-ray diffraction (XRD), Raman spectroscopy, X-ray photoelectron spectroscopy (XPS), transmission electron microscopy (TEM), and high-resolution transmission electron microscopy (HRTEM) and energy-dispersive X-ray spectroscopy (XDS). The characterization results showed that CeO_2_-NRs, CeO_2_-NCs, and CeO_2_-NPs mainly expose (110), (100) and (111) facets, respectively. Moreover, CeO_2_-NRs and CeO_2_-NCs present higher oxygen vacancy concentration than CeO_2_-NPs. In the CO_2_ reforming of methane reaction, Ru/CeO_2_-NR and Ru/CeO_2_-NC catalysts showed better catalytic performance than Ru/CeO_2_-NPs, indicating that the catalysts with high oxygen vacancy concentration are beneficial for promoting catalytic activity.

## 1. Introduction

As one of the most important emissions of modern industry and life, CO_2_ has attracted great attention for its influence on global warming [1,2,3]. Many efforts have been made by governments around the world to depress the emission of CO_2_ [4,5]. Actually, the use of CO_2_ as a type of C1 resource for further chemical industry is also an efficient way to solve the problem [5,6]. As another important greenhouse gas, CH_4_ exists extensively in natural resources. However, the current obstacle of the application of CH_4_ is the activation of CH_4_ due to its stable structure [7,8,9]. Thus, the studies of various reforming reactions have important industrial values and scientific significance in the application of CH_4_. Dry reforming of methane attracted more attention in recent years because this reaction could eliminate both greenhouse gases, CO_2_ and CH_4_, simultaneously. Furthermore, the products H_2_ and CO (syngas) of the reaction have a low H_2_/CO molar ratio of nearly 1, which is more suitable for the subsequent chemical industrial processes [10,11,12].

For the methane dry reforming reaction, many kinds of catalysts have been widely studied by loading active metals on different supports. Ni, Co, Rh, Ru, Pd, Ir, and Pt have been studied for methane dry reforming reaction in recently years [7,13,14,15]. Ni- and Co-based catalysts attracted more interest due to their lower price than noble metals. However, the sintering and coking problems along with the poor activity of the catalysts at relatively low temperature are difficult to solve. As a result, the study of noble metals in methane dry reforming is of great significance and the Rh- or Ru-based catalysts have been considered to have better activity and stability [15,16,17]. On the other hand, a lot of work has focused on the influence of the different metal oxide supports, such as SiO_2_, γ-Al_2_O_3_, MgO, ZrO_2_, La_2_O_3_, and TiO_2_ [18,19,20,21,22,23,24]. These studies showed that the supports played an important role in their catalytic activity. The supports may not only offer various textures to disperse the active metal components, but also interact strongly with the active species. The interaction between supports and active metals influence on the structure of catalysts, the particle sizes, and the dispersion of active sites consequently influences the catalytic activity of the catalysts [25]. For example, Wang et al. found that the catalytic performance of a series of catalysts for methane dry reforming reaction was related to the redox properties of the supports [26].

CeO_2_ is a unique metal oxide due to its low redox potential and high oxygen vacancy mobility, which could have a strong interaction with the active metals [27,28,29]. In recent years, CeO_2_ has been widely used as a catalyst promoter or support for various reactions, such as dry reforming of methane, partial oxidation of methane, dry reforming of propane, and steam reforming of ethanol [30,31,32,33,34]. The studies indicate that the existence of CeO_2_ in catalysts benefits their resistance to carbon deposition, because the coke on the catalyst could be removed by reacting with the oxygen species on the surface of CeO_2_. Thus, the release of oxygen species is a crucial process for CeO_2_-based catalysts. Generally, the surface energy of metal oxides is strongly related with their microstructures, and there are evident differences in surface energy among the faces with various indices of the crystallographic plane. The catalytic activity of shape-dependent CeO_2_-supported active metal is obviously different [35,36,37,38,39,40]. Therefore, it is reasonable to believe that the use of CeO_2_ supports specific exposed crystal facets may influence the catalytic behavior of the catalyst in dry reforming of methane.

In this work, we have prepared a series of CeO_2_ nanorods (CeO_2_-NRs), nanocubes (CeO_2_-NCs), and nanopolyhedra (CeO_2_-NPs) with dominant specific crystal facets (110), (100), and (111), respectively. These morphology-controlled CeO_2_ were used as supports for Ru as a catalytic active component through precipitation and deposition methods. The catalysts were characterized, and their catalytic behavior for dry reforming of methane was studied. In particular, the influence of the exposed crystal facets of the CeO_2_ supports on the catalytic activities was investigated in detail.

## 2. Results and Discussion

### 2.1. Catalyst Characterization

#### 2.1.1. TEM and High-Resolution Transmission Electron Microscopy (HRTEM) Analysis

**Figure 1:**
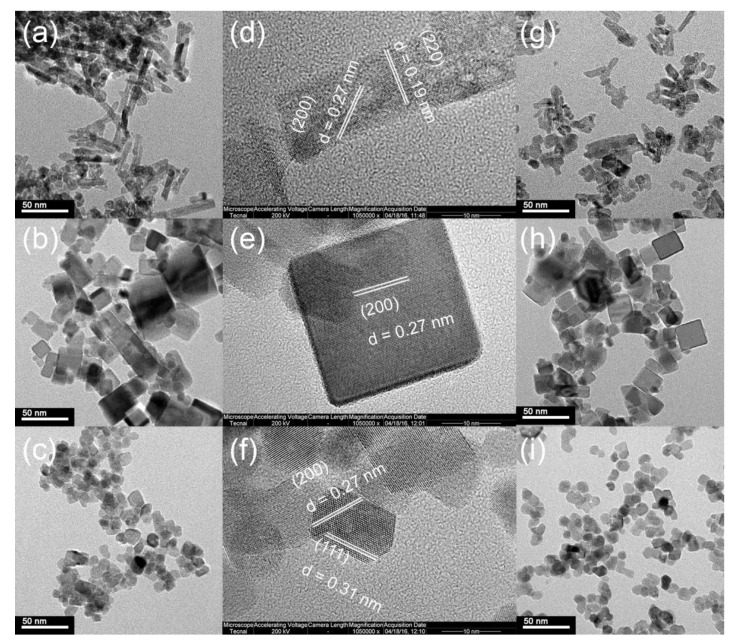
TEM images of (**a**) CeO_2_-NRs, (**b**) CeO_2_-NCs, and (**c**) CeO_2_-NPs; high-resolution transmission electron microscopy (HRTEM) images of (**d**) CeO_2_-NRs, (**e**) CeO_2_-NCs, and (**f**) CeO_2_-NPs; TEM images of (**g**) Ru/CeO_2_-NRs, (**h**) Ru/CeO_2_-NCs, and (**i**) Ru/CeO_2_-NPs.

Figure 1 shows the TEM and HRTEM images of the CeO_2_ supports and Ru/CeO_2_ catalysts. In Figure 1a–f, three CeO_2_ samples show different morphologies. CeO_2_-NRs with the rod-like morphology in Figure 1a,d exhibit a uniform diameter of ca. 10 nm with two obvious interplanar spacings of 0.19 and 0.27 nm, corresponding to the (220) and (200) lattice fringes, respectively. The shape of CeO_2_-NRs indicates that the nanorods grow along the (110) direction. Figure 1b,e shows that CeO_2_-NCs possess the cubic morphology with a size of around 10–50 nm and clear (200) lattice fringes with the interplanar spacing of 0.27 nm. Figure 1c,f shows CeO_2_-NPs in the size range of 10–20 nm and (111) and (200) lattice fringes corresponding to the interplanar spacing of 0.31 and 0.27 nm, respectively [41]. HRTEM results show that only the (100) facet exists in the CeO_2_-NCs, whereas the (110) and (111) facets are the mainly exposed planes for CeO_2_-NRs and CeO_2_-NPs, respectively. Figure 1g–i shows TEM images of 3% Ru/CeO_2_ catalysts. It can be seen that the morphology of the supports remains after the loading of Ru species and no large aggregation was observed, indicating the high dispersion of the Ru species. The distribution of Ce and Ru was evaluated by EDS analysis. The EDS results are shown in Figures S1–S3 for the Ru/CeO_2_-NRs, Ru/CeO_2_-NCs, and Ru/CeO_2_-NPs, respectively. The elemental mapping results also indicate that Ru species are highly dispersed on the surface of CeO_2_ supports.

#### 2.1.2. X-ray Diffraction

**Figure 2:**
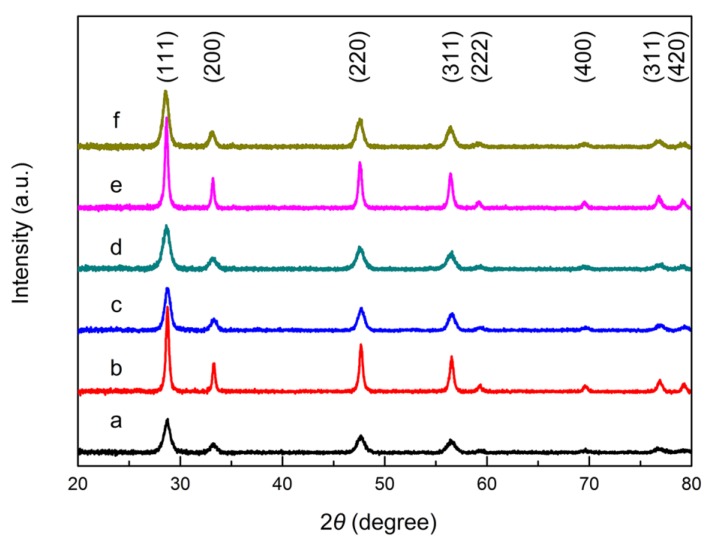
XRD patterns of (a) CeO_2_-NRc, (b) CeO_2_-NCs, (c) CeO_2_-NPs, (d) Ru/CeO_2_-NRs, (e) Ru/CeO_2_-NCs, and (f) Ru/CeO_2_-NPs.

Figure 2 shows the XRD patterns of CeO_2_ samples with different exposed facets and their corresponding 3% Ru/CeO_2_ catalysts. The peaks of CeO_2_-NRs, CeO_2_-NCs, and CeO_2_-NPs at 28.7°, 33.2°, 47.6°, 56.5°, 59.1°, 69.4°, 76.6°, and 79.2° are assigned to the diffractions of (111), (200), (220), (311), (222), (400), (311), and (420) of CeO_2_ indexed to the face-centered cubic fluorite structure with space group *Fm-3m* (JCPDS 34-0394). The crystalline sizes of different samples calculated by Scherrer formula are listed in Table 1. The results are consistent with TEM images. The diffraction peak positions of the three CeO_2_ supports are nearly the same, but their relative intensities are different. The different ratios of (I_(200)_/I_(111)_ + I_(220)_/I_(111)_) as 1.00, 1.02, and 0.82 for CeO_2_-NRs, CeO_2_-NCs, and CeO_2_-NPs, respectively, show that the (111) facet exposure in the nanopolyhedra is higher than that in nanorods and nanocubes, which are consistent with the TEM and HRTEM analysis [39]. The XRD patterns of different Ru/CeO_2_ catalysts are shown in Figure 2d–f, and no significant change of the diffractions of CeO_2_ can be found, suggesting that the structure of CeO_2_ supports remains. It is worth to note that from the inductively coupled plasma atomic emission spectrometer (ICP-AES) results (Table 1), Ru/CeO_2_ samples show close Ru loading to the feeding value of 3 wt.% despite some weight loss. No new diffraction assigned to Ru species appears in the XRD patterns, indicating the high dispersion of metal species on the support. The Ru dispersion calculated from CO-TPD shows that the Ru species are highly dispersed on the CeO_2_ supports. Ru/CeO_2_-NRs and Ru/CeO_2_-NCs display higher Ru dispersion (54% and 59%, respectively) than that of Ru/CeO_2_-NPs (35.0%) (Table 1). The results are consistent with the EDS mappings results (Supplementary Materials).

#### 2.1.3. N_2_ Adsorption–Desorption

The N_2_ adsorption–desorption characterization was performed at 77 K to study the textural properties of CeO_2_ supports and Ru/CeO_2_ catalysts. The Brunauer–Emmett–Teller (BET) surface areas of CeO_2_-NRs, CeO_2_-NCs, and CeO_2_-NPs and their corresponding Ru-supported catalysts, Ru/CeO_2_-NRs, Ru/CeO_2_-NCs, and Ru/CeO_2_-NPs are 87.1, 31.7, 67.4, 84.9, 30.1, and 65.8 m^2^·g^−1^, respectively. As shown in Table 1, the surface areas of pure CeO_2_-NRs and CeO_2_-NPs are higher than those of CeO_2_-NCs, which is mainly because of smaller particle sizes of the former compared to the latter. The surface areas and particle sizes are merely changed after the addition of Ru species with low loading. It is well known that the surface area has an influence on the catalytic activity for many reactions, and the catalysts with a large surface area would be beneficial to enhance the catalytic activity. However, for some reaction over ceria-based catalysts, the surface area is not the crucial factor based on the previous research [33,36].

#### 2.1.4. H_2_-Temperature Programmed Reduction

**Figure 3:**
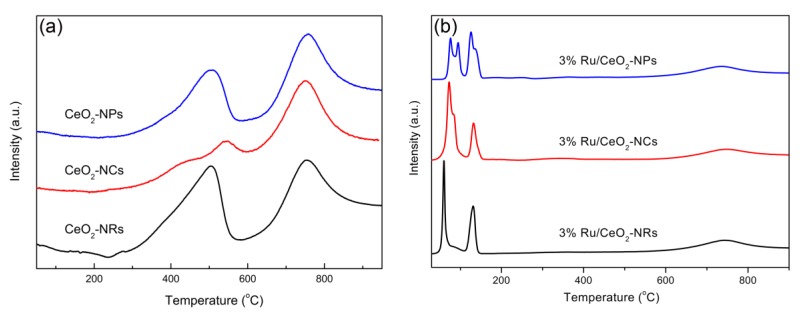
H_2_-temperature programmed reduction (H_2_-TPR) curves of (**a**) CeO_2_ samples (**b**) Ru/CeO_2_ samples.

The H_2_-temperature programmed reduction (H_2_-TPR) measurements were performed to clarify the reduction characteristics of CeO_2_ supports and supported Ru/CeO_2_ catalysts. As shown in Figure 3a, two reduction peaks appear in the temperature range of 250–900 °C for three CeO_2_ supports with different exposed facets. The first peak below 600 °C is due to the reduction of Ce^4+^ to Ce^3+^ on the CeO_2_ surface with oxygen vacancy, and the second peak is the reduction of Ce^4+^ to Ce^3+^ inside the bulk CeO_2_ [27,40]. After loading the Ru species on the supports, the reduction peak changed significantly. The presence of two sets of peaks at 30–110 °C and 110–200 °C indicates Ru species exist in two different states. The reduction peaks at low temperature are usually assigned to the adsorbed oxygen and well-dispersed Ru species interacting strongly with the CeO_2_ supports [42]. From the previous research, the surface energies (γ) associated with different crystallographic planes are usually different, and a general sequence is γ(111) < γ(100) < γ(110) [43,44]. Thus, the order of reduction temperature of all catalysts in the range of 30–110 °C is Ru/CeO_2_-NRs < Ru/CeO_2_-NCs < Ru/CeO_2_-NPs, which agrees with the energy order of different facets. The reduction peaks at a relatively high temperature are assigned to the Ru species interacting weakly with CeO_2_ supports and the surface oxygen of CeO_2_. It has been reported that the hydrogen consumption is 178–188 μmol·g^−1^ for the reaction of RuO_2_ + 2H_2_ → Ru^0^ + 2H_2_O [42]. The total H_2_ consumption calculated by integrating the peaks is ~754 μmol·g^−1^ for Ru/CeO_2_-NRs, ~710 μmol·g^−1^ for Ru/CeO_2_-NCs, and ~638 μmol·g^−1^ for Ru/CeO_2_-NPs. The high hydrogen consumption of three catalysts should result from the reduction of a large amount of surface oxygen of CeO_2_ due to the existence of Ru–O–Ce. The low hydrogen consumption of Ru/CeO_2_-NPs indicates that the Ru species have a weak interaction with CeO_2_ support compared to those of Ru/CeO_2_-NRs and Ru/CeO_2_-NCs.

#### 2.1.5. Raman Spectroscopy

**Figure 4:**
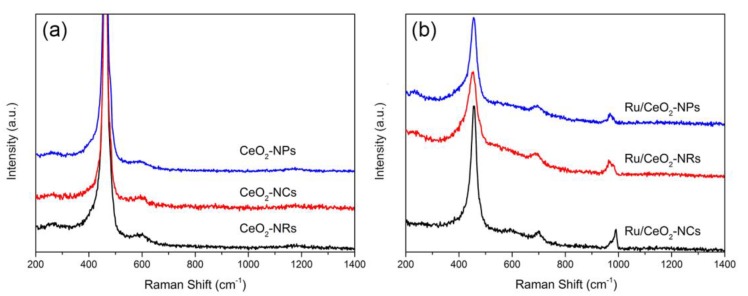
Raman spectra of (**a**) CeO_2_ samples (**b**) Ru/CeO_2_ samples.

Figure 4 shows Raman spectra of CeO_2_ supports and Ru/CeO_2_ catalysts. All three CeO_2_ supports with different morphologies exhibit a strong peak at around 461 cm^−1^, which is assigned to the vibration model (F_2g_) of the CeO_2_ fluorite phase. Additionally, the other three weak peaks at 257, 594, and 1170 cm^−1^ are assigned to second-order transverse acoustic (2TA) mode, defect-induced (D) mode, and second-order longitudinal optical (2LO) mode, respectively [35,36,37,38,45]. The relative intensity of I_(594+1170)_/I_461_ reflects the intrinsic concentration of defect sites on CeO_2_ supports, such as oxygen vacancies [38,46]. The calculation results show that the concentration of vacancy sites decreases in the following order of CeO_2_-NRs > CeO_2_-NCs > CeO_2_-NPs (Table 2). This is because the formation energy of vacancy is in a reversed order of surface energies on different facets [43]. After the loading of the Ru species, two new peaks appear at 694 and 968 cm^−1^ in addition to the peaks of CeO_2_ supports, which are ascribed to the formation of the Ru–O–Ce bond between metal oxides and supports [38,40]. The relative intensity ratio of I_(694+968)_/I_461_ implies that the interaction between Ru and CeO_2_ supports on Ru/CeO_2_-NR and Ru/CeO_2_-NC samples is stronger than that on the Ru/CeO_2_-NP sample [38], in accordance with the TPR results.

#### 2.1.6. X-ray Photoelectron Spectroscopy (XPS)

**Figure 5:**
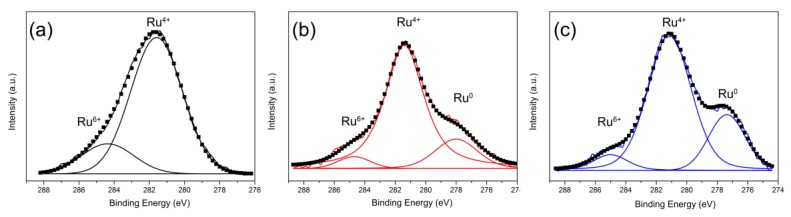
Ru 3d X-ray photoelectron spectroscopy (XPS) spectra of (**a**) Ru/CeO_2_-NRs, (**b**) Ru/CeO_2_-NCs, and (**c**) Ru/CeO_2_-NPs.

Figure 5 shows Ru 3d XPS spectra of Ru/CeO_2_ samples before H_2_ reduction. The Ru XPS results of Ru/CeO_2_-NCs and Ru/CeO_2_-NPs show three peaks around 277.5, 281.4, and 284.8 eV, which are assigned to Ru^0^, Ru^4+^, and Ru^6+^, respectively. The Ru/CeO_2_-NRs only exhibit two Ru species, which could be assigned to Ru^6+^ and Ru^4+^ [35,38]. Moreover, the content of Ru^4+^ follows the order: Ru/CeO_2_-NRs > Ru/CeO_2_-NCs > Ru/CeO_2_-NPs (Table 3). The Ru^4+^ ions may insert into the surface lattice of CeO_2_ to increase of oxygen vacancy concentration on the CeO_2_ support [38]. This effect leads to the high concentration of surface oxygen vacancies for Ru/CeO_2_-NRs and Ru/CeO_2_-NCs catalysts, and, on the other hand, the decrease of the aggregation of RuO_2_ particles.

**Figure 6:**
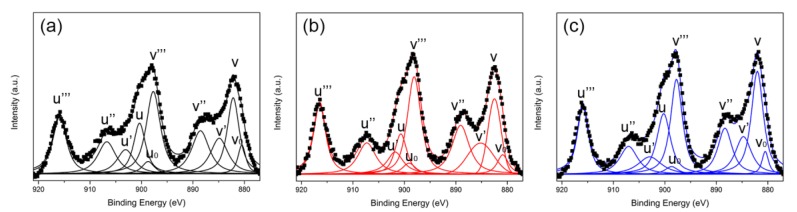
Ce 3d XPS spectra of (**a**) Ru/CeO_2_-NRs, (**b**) Ru/CeO_2_-NCs, and (**c**) Ru/CeO_2_-NPs.

Figure 6 shows the Ce 3d XPS spectra of three Ru/CeO_2_ samples. There are ten peaks resulting from the pairs of spin orbit doublets, which can be identified through deconvolution. The four peaks around 880.6 (v_0_), 884.4 (v′), 898.8 (u_0_), and 901.0 eV (u′) are assigned to Ce^3+^ species, and six peaks around 882.2 (v), 888.6 (v″), 898.3 (v‴), 900.7 (u), 907.7 (u″), and 916.2 eV (u‴) are assigned to Ce^4+^ species [36,37,38,47]. The intensity ratio of Ce^3+^/(Ce^3+^ + Ce^4+^) is 22.8%, 21.1%, and 19.8% for Ru/CeO_2_-NRs, Ru/CeO_2_-NCs, and Ru/CeO_2_-NPs, respectively. The appearance of Ce^3+^ species leads to the formation of oxygen vacancy on the CeO_2_ surface. The high concentration of Ce^3+^ on the surface reflects the high concentration of surface oxygen vacancies, which benefits the activation and conversion of reactants in dry reforming of methane reaction [40].

**Figure 7:**
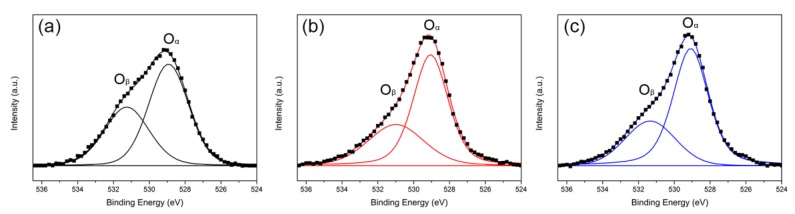
O 1s XPS spectra of (**a**) Ru/CeO_2_-NRs, (**b**) Ru/CeO_2_-NCs, and (**c**) Ru/CeO_2_-NPs.

Figure 7 shows O 1s XPS spectra of Ru/CeO_2_ samples. All three samples mainly exist in two peaks corresponding to two kinds of oxygen species. The peak around 529 eV is ascribed to the oxygen species inside of the CeO_2_ lattice, marked as the O_α_ species, and the peak around 531 eV marked O_β_ species is mainly ascribed to low coordination oxygen defects on the surface [36,48]. As shown in Table 3, Ru/CeO_2_-NRs have the highest O_β_/O_α_ ratio and Ru/CeO_2_-NPs have the lowest O_β_/O_α_ ratio. The O_β_/O_α_ ratio estimated through deconvolution shows that the content of surface oxygen defects on CeO_2_ is morphology-dependent.

### 2.2. Catalytic Performance for the Dry Reforming of Methane

**Figure 8:**
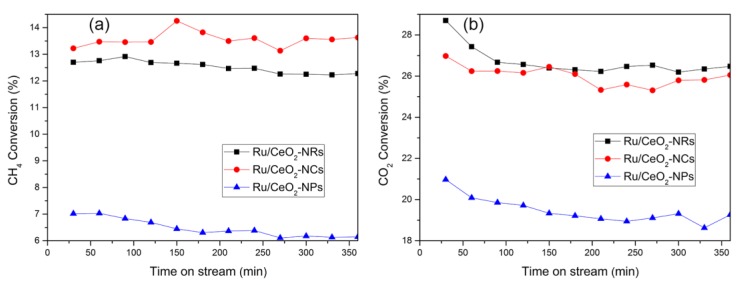
The CH_4_ conversion (**a**) and CO_2_ conversions (**b**) of three Ru/CeO_2_ samples versus time at 500 °C.

**Figure 9:**
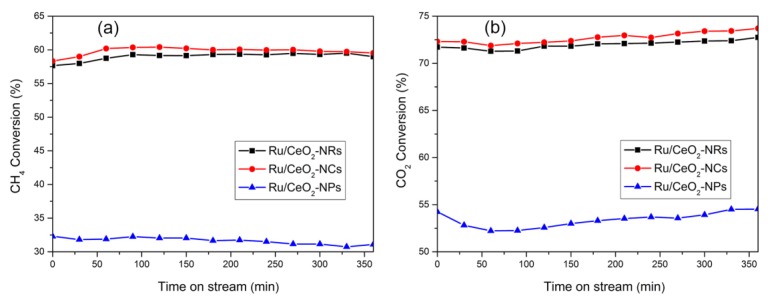
The CH_4_ conversion (**a**) and CO_2_ conversions (**b**) of three Ru/CeO_2_ samples versus time at 650 °C.

The catalytic performance of three CeO_2_-supported Ru catalysts in the dry reforming of methane under the reaction temperature of 500 °C and 650 °C is shown in Figure 8 and Figure 9. Compared to high CO_2_ conversion, low CH_4_ conversion is due to the simultaneous occurrence of a reverse water–gas shift reaction. Moreover, the catalytic activities for all three catalysts remain relatively stable after a 240-min reaction. The conversion trends of CO_2_ and CH_4_ are followed by Ru/CeO_2_-NRs (26.5% of CO_2_ and 12.3% of CH_4_) ≈ Ru/CeO_2_-NCs (26.0% of CO_2_ and 13.6% of CH_4_) > Ru/CeO_2_-NPs (19.3% of CO_2_ and 6.2% of CH_4_) after a 240-min reaction process. Both CO_2_ and CH_4_ conversions increase for all three catalysts when increasing the temperature to 650 °C (Figure 9). The conversions of CO_2_ and CH_4_ over Ru/CeO_2_-NR and Ru/CeO_2_-NC samples are much higher than those for Ru/CeO_2_-NPs.

From the above results, CeO_2_ exposed with a high energy facet of (110) and (100) have a high catalytic activity in the methane dry reforming reaction. This is due to the strong interaction between Ru species and supports exposed (110) and (100) facets, and the (110) and (100) facets on the CeO_2_ with high oxygen vacancy concentration benefit the activation of CO_2_ during the reaction.

## 3. Experimental Section

### 3.1. Preparation of CeO_2_ Samples

Morphology-controlled CeO_2_ samples were synthesized using the hydrothermal method. For the preparation of CeO_2_-NCs and CeO_2_-NPs, 1.82 g of Ce(NO_3_)_3_·6H_2_O was dissolved in 40 mL of distilled water and 16.8 g of NaOH for CeO_2_-NCs or 0.33 g of NaOH for CeO_2_-NPs were dissolved in 30 mL of distilled water. The Ce(NO_3_)_3_ solution was added into the NaOH solution dropwisely under stirring and stirred for further 30 min to form a milky slurry. Then, the milky slurry was transferred into a 100-mL stainless steel autoclave and hydrothermally treated at 180 °C for 24 h. After the autoclave was cooled to room temperature naturally, the precipitates were separated by centrifugation, washed with distilled water thoroughly, dried at 60 °C for 12 h, and calcined at 500 °C with a heating rate of 1 °C min^−1^ from room temperature for 4 h to obtain the CeO_2_-NCs and CeO_2_-NPs. The synthesis procedure of CeO_2_-NRs was the same as that of CeO_2_-NCs, except that the hydrothermal treatment temperature was 100 °C.

### 3.2. Preparation of Ru/CeO_2_ Catalysts

The Ru/CeO_2_ catalysts were prepared using the precipitation and deposition method. A total of 1.0 g of CeO_2_ was added into a 20 mL of solution containing 0.015 g of RuCl_3_·3H_2_O and stirred for 30 min. Then, 0.1 M NH_3_·H_2_O aqueous solution was added to adjust the pH of the suspension up to 8.0, and then the suspension was aged at room temperature for 3 h. The catalyst in suspension was separated by centrifugation, washed with distilled water, dried at 60 °C for 12 h, and calcined at 500 °C with a heating rate of 1 °C ·min^−1^ from room temperature for 4 h. The loading of metal Ru in the catalysts is 3 wt.%.

### 3.3. Catalyst Characterization

Transmission electron microscopy (TEM) and high-resolution TEM (HRTEM) images were obtained on a JEM-2011 transmission electron microscope (JOEL, Japan) and a FEI Tecnai G^2^ F20 S-Twin field-emission transmission electron microscope (Hillsboro, OR, USA, respectively. X-ray diffraction (XRD) patterns were obtained on a Bruker D8 Advance diffractometer (Karlsruhe, Germany), using Cu K*α* radiation (*λ* = 1.5418 Å) at 40 kV and 40 mA, a scanning rate of 5^o^·min^−1^, a step size of 0.02°, and a 2*θ* angle ranging from 20° to 80°. The N_2_ adsorption–desorption characterization was performed using a Micromeritics Tristar 3000 apparatus (Quantachrome, Boynton Beach, FL, USA). Prior to the adsorption measurements, the sample was outgassed at 250 °C for 3 h under vacuum. The specific surface areas were calculated using the Brunauer–Emmett–Teller (BET) method. Elemental analysis was performed on a Thermo Elemental IRIS Intrepid inductively coupled plasma atomic emission spectrometer (ICP-AES, Thermo Elemental, Waltham, MA, USA). X-ray photoelectron spectroscopy (XPS) was performed on a Versa Probe PHI 5000 instrument with Al Kα radiation (Versa Probe, Amreica). The binding energies were calibrated using the containment carbon (C1s = 284.6 eV). H_2_-temperature programmed reduction (H_2_-TPR) was performed using a Micromeritics ChemiSorb 2720 apparatus with a thermal conductivity detector (TCD, Micromeritics, Chanhassen, MN, USA). Before measurement, 45.0 mg of sample was placed in a U-shape quartz tube and degassed under flowing He at 200 °C for 2 h, cooled to room temperature, and then switched gas to 10% H_2_/Ar. The sample was reduced in a stream of 10% H_2_/Ar (50 mL·min^−1^) with a heating rate of 10 °C min^−1^ from room temperature up to 900 °C. CO-temperature programmed desorption (CO-TPD) was performed to determine the dispersion of Ru particles using a Micromeritics ChemiSorb 2720 apparatus with TCD (Micromeritics, Chanhassen, MN, USA). The Ru:CO molar ratio in the chemisorption was taken as 1 [49]. The sample (45.0 mg) was firstly reduced in a stream of 10% H_2_/Ar (50 mL·min^−1^) at 500 °C for 2 h. Subsequently, the reduced sample was purged in He (50 mL min^−1^) at 500 °C for 30 min to remove excess H_2_ and then cooled down to 40 °C for adsorption of CO for 30 min, and then gas was switched to He to keep 30 min at 40 °C to remove excess CO. Finally, the samples were heated in He with a temperature ramp of 10 °C min^−1^.

### 3.4. Catalytic Activity Measurements

The catalytic measurements for dry reforming of methane were carried out in a quartz tube with the inner diameter of 6 mm using a fixed-bed reactor system at atmospheric pressure. The gas flow rate was controlled by mass-flow controllers. A total of 30 mg of 40–60 mesh catalyst was mixed with 250 mg of 40–60 mesh inert quartz sand and placed into the reactor. Before the reaction, the catalyst was reduced in a flow of H_2_ (30 mL·min^−1^) at 500 °C for 2 h. Then, the reaction gas mixture consisting of CO_2_, CH_4_, and N_2_ (CO_2_:CH_4_:N_2_ volume ratio of 1:1:3) was introduced into the reaction with the gas hourly space velocity (GHSV) of 24,000 mL·h^−1^·g^−1^ (CO_2_ + CH_4_ + N_2_), and the effluent product gases were cooled in an ice-water bath and analyzed by online gas chromatography with a thermal conductivity detector (TCD) using a TDX-01 packed column. The reaction activity of the methane dry reforming reaction was tested at 500 °C and 650 °C.

## 4. Conclusions

In this work, a series of CeO_2_ supports, CeO_2_-NRs, CeO_2_-NCs, and CeO_2_-NPs, with different exposed facets were synthesized via the hydrothermal method; the metal Ru was loaded on the CeO_2_ supports as catalyst for the dry reforming of methane. The CeO_2_-NR and CeO_2_-NC supports with mainly (110) and (100) exposed facets contain higher oxygen vacancy concentration than the CeO_2_-NP supports with (111) exposed facets. The high energy surface structure of the CeO_2_-NRs and CeO_2_-NCs enhances the interaction between Ru and CeO_2_. The catalytic results of the three catalysts for dry reforming of methane are related with the surface energy of the CeO_2_ supports and the Ru/CeO_2_-NR and Ru/CeO_2_-NC catalysts perform a higher catalytic activity than Ru/CeO_2_-NPs, due to the former containing higher oxygen vacancy concentration.

## Figures and Tables

**Table 1:** Physical properties of various samples.

Sample	Specific Surface Area ^a^ (m^2^·g^−1^)	Particle Size ^b^ (nm)	Mean Sizes ^c^ (nm)	Ru ^d^ (wt.%)	Ru Dispersion ^e^ (%)	
CeO_2_-NRs	87.1	(8 ± 2) × (50 − 100)	13.6	-		
CeO_2_-NCs	31.7	26 ± 15	23.0	-		
CeO_2_-NPs	67.4	13 ± 5	13.9	-		
Ru/CeO_2_-NRs	84.9	(7 ± 3) × (20 − 100)	12.1	2.4	54	
Ru/CeO_2_-NCs	30.1	24 ± 15	23.6	2.4	59	
Ru/CeO_2_-NPs	65.8	12 ± 5	13.7	2.3	35	

^a^ Surface area determined from N_2_ isotherm. ^b^ Calculated for about 100 nanoparticles from the TEM images. ^c^ Estimated by Scherrer equation, applied to the (111) reflection on fluorite CeO_2_. ^d^ Analyzed by inductively coupled plasma atomic emission spectrometer (ICP-AES). ^e^ calculated based on the CO-TPD results.

**Table 2:** Raman spectral data of CeO_2_ and Ru/CeO_2_ samples.

Sample	I_(__594__+ 117__0__)_/I_46__1_	I_(69__4__+ 9__68__)_/I_46__1_
CeO_2_-NRs	0.116	-
CeO_2_-NCs	0.102	-
CeO_2_-NPs	0.057	-
Ru/CeO_2_-NRs	-	0.234
Ru/CeO_2_-NCs	-	0.187
Ru/CeO_2_-NPs	-	0.177

**Table 3:** XPS data of Ru/CeO_2_-NR, Ru/CeO_2_-NC, and Ru/CeO_2_-NP samples.

Samples	Ru^4+^ (%)	Ce^3+^/(Ce^3+^ + Ce^4+^) (%)	O_β_/O_α_
Ru/CeO_2_-NRs	80.9	22.8	0.40
Ru/CeO_2_-NCs	78.9	21.1	0.36
Ru/CeO_2_-NPs	22.4	19.8	0.32

## Supplementary Materials

The following are available online, Figure S1: Ce, Ru, and O mapping with EDS spectrum of the Ru/CeO_2_-NR catalyst. Figure S2: Ce, Ru, and O mapping with EDS spectrum of the Ru/CeO_2_-NC catalyst. Figure S3: Ce, Ru, and O mapping with EDS spectrum of the NRu/CeO_2_-NP catalyst.
